# Changes in Phenolic Compounds and Antioxidant Activity of Fruit Musts and Fruit Wines during Simulated Digestion

**DOI:** 10.3390/molecules25235574

**Published:** 2020-11-27

**Authors:** Tomasz Tarko, Aleksandra Duda-Chodak, Agata Soszka

**Affiliations:** Department of Fermentation Technology and Microbiology, Faculty of Food Technology, University of Agriculture in Krakow, ul. Balicka 122, 30-149 Krakow, Poland; a.duda-chodak@ur.krakow.pl (A.D.-C.); agata.soszka@gmail.com (A.S.)

**Keywords:** fruit wines, in vitro digestion, polyphenols, antioxidant activity

## Abstract

The content of polyphenols (total phenolic content (TPC)) and the antioxidant activity (AOX) of food products depend on the raw materials used and the technological processes in operation, but transformations of these compounds in the digestive tract are very important. The aim of this study was to determine the TPC, profile of polyphenols, and AOX of apple and blackcurrant musts and wines in order to evaluate the changes occurring in a simulated human digestive system. The research material consisted of apples and blackcurrant, from which musts and fruit wines were obtained. All samples were subjected to three-stage digestion in a simulated human digestive system and then analyzed for the following: TPC (Folin–Ciocalteu method) and profile (HPLC), AOX (method with 2,2′-azino-bis (3-ethylbenzothiazoline-6-sulfonic) acid (ABTS) radical), and for the wines also total extract, volatile acidity (International Organization of Vine and Wine (OIV) method), and sugar profile (HPLC). The antioxidant activity of fruit wines is directly related to the total polyphenol content. Phenolic compounds were transformed during all digestive stages, which led to the formation of compounds with higher antioxidant capacity. The largest increase in polyphenols was observed after the digestive stage in the small intestine. Transformations of phenolic compounds at each digestive stage resulted in the formation of derivatives with higher antioxidant potential.

## 1. Introduction

Metabolites of biologically active compounds formed during digestion that then reach the blood and target organs often have different biological activity compared to primary compounds [1]. These phenolic compounds, which occur in the human diet in the largest quantities, do not always show the highest biological activity after consumption; this may be due to their limited absorption in the digestive tract, intensive metabolism to derivatives with lower activity, or their rapid elimination (degradation). For example, the antioxidant activity (AOX) of quercetin glycosides is half that of aglycon [2].

Individual groups of polyphenols differ significantly in bioavailability, which is associated with their structure, molecular weight, polarity, and form. The metabolic reaction of most dietary polyphenols has several similarities as follows: (1) glycosides of these compounds undergo hydrolysis prior to absorption; (2) mainly glucuronates and sulfates of native particles are present in the plasma; (3) in the polyphenols containing hydroxyl groups in the ortho position, methylation may occur; (4) and aglycons are either absent in the bloodstream or present in small quantities (except for catechins derived from green tea) [3,4]. The highest number of metabolic transformations and absorption of polyphenols occurs in the small intestine, but the course of this digestive stage differs for individual flavonoid groups [5,6,7,8]. Polyphenols that have not been absorbed in the small intestine, and those that have been secreted through bile or directly from enterocytes due to metabolic processes, reach the large intestine. In this part of the digestive tract, intestinal microbiota resides, which has extremely diverse enzymatic activity. Because of bacterial enzymes, a large number of reactions occur in the large intestine, including deconjugation, dehydroxylation, demethylation, isomerization, decarboxylation, hydrolysis of various chemical bonds, and cleavage of aromatic rings, resulting in a mixture of simple phenolic acids [9].

Edible fruits differ in the content of phenolic compounds in their tissues. Some of them are very rich in antioxidants, e.g., chokeberry, blackcurrant, and wild rose. Others, such as apples, do not contain significant amounts of phenolic compounds; however, due to the frequency or amount in which they are consumed, they are an important source of these ingredients from a nutritional point of view.

Apples are one of the most common fruits in the world due to their taste and the ability to obtain a wide range of processed products (juices, purees, wines, and confectionery additives). Fruits contain polyphenols from various groups, including procyanidins, phenolic acids, dihydrochalcones, flavan-3-ols, flavonols, and anthocyanins, but their concentration is not high and strongly depends on which part of the plant they are taken from, fruit variety, climatic conditions, and many other factors [10,11,12,13]. In addition, the polyphenol content decreases when processing apples [14]. Apples are one of the most commonly used raw materials in fruit winemaking, due to their availability, price, and chemical composition, which allow to obtain a high-quality product with good sensory qualities.

Blackcurrant, which belongs to the gooseberry family, is grown mainly in Europe, and is primarily used for the production of nectars, syrups, jams, and wines, as well as ingredients for juices and non-alcoholic beverages. Blackcurrant contains a number of phenolic compounds and is characterized by high antioxidant activity; this increases the possibility of its use in the functional food sector [15]. Blackcurrants are a good source of vitamins (A, C, E, and folic acid), provitamins (carotene and lutein), mineral compounds (calcium and selenium), and phytosterols; however, their health-promoting properties are primarily associated with the presence of phenolic compounds (anthocyanins, flavonols, flavan-3-ols, proanthocyanidins, soluble tannins, and phenolic acids) as the main bioactive components of fruits [15,16]. Regardless of how the fruit is processed, there is always a reduction in polyphenols of up to 80% in the final product [17]; this is associated with a high content of pectins in the skin cell wall making it difficult to extract these compounds. Despite this, among the berry fruit juices, blackcurrant juice has the highest content of phenolic compounds and the best ability to quench free radicals [18]. Blackcurrants are a valued raw material for wine due to their characteristic strong aroma and taste, but the high acidity of this fruit makes it necessary to deacidify the musts by dilution or by the biological method [19].

The processing of fruit into juices and wines is very popular; however, during enzymatic treatment, pressing, clarification, and other technological processes, as well as fermentation itself, there is a transformation of phenolic compounds, which significantly affects the final content and profile of polyphenols in beverages. In addition, the different chemical composition of beverages is associated with the different bioavailability of these valuable ingredients during digestion. The bioavailability of polyphenolic compounds from lyophilized currant juice has already been described previously [20], but the authors focused mainly on whether the anti-inflammatory effects of blackcurrant fruits could be modulated by metabolic transformations. The bioavailability of polyphenols from apple juice was also analyzed [21]. However, there are still no publications comparing the bioavailability of polyphenolic compounds from fruit musts and the wines obtained from them. Therefore, for this study, musts were made from apples and blackcurrants, and then they were subjected to ethanol fermentation to obtain wines. The aim of the study was to assess the content and profile of phenolic compounds and antioxidant activity at various stages of digestion of wines and musts in a simulated human digestive tract.

## 2. Results and Discussion

### 2.1. Characteristics of Wines

Both wines obtained had an alcohol content of 12% vol. (Table 1) that, according to Polish guidelines, classifies them as medium wines [22]. Ethanol concentration is directly related to the content of fermenting sugars in the setting, the temperature of the fermentation process, and the yeast strain used [23]. The total extract contains compounds remaining in the wine after distilling off the alcohol and other volatile substances. These include residual fermentation sugars, tannins, tannic acids, and many more compounds [24]. In the obtained wines, the content of these compounds was between 20 g/L and 22 g/L (Table 1).

Total acidity and volatile acidity are important quality determinants of wines. Moreover, in this respect, the obtained fruit wines fall within satisfactory limits (Table 1), with the total acidity of the wine, expressed as malic acid, being in the range from 3.5 g/L to 9 g/L, while the volatile acidity, expressed as acetic acid, did not exceed 1.3 g/L [25]. The acid content significantly affects the taste of wine, giving it a refreshing character and reducing perceptible sweetness. A suitably low pH value is also crucial for the color stability of red fruit wines rich in anthocyanins, and it also prevents the oxidation of phenolic compounds. The higher acidity of wines also provides an antimicrobial effect [26]. In studies conducted by Hecke et al. [27] on eighteen apple varieties, in terms of quantity malic acid dominated, the presence of shikimic and citric acids was also found. The high total acidity of blackcurrant is affected by a significant content of citric, malic, and tartaric acids [28]. The results obtained in these studies were comparable with the total acidity determined in thirteen varieties of these fruits [15].

The apple wines examined in this work contained less than 10 g/L of sugars, which classifies them as dry fruit wines, whereas the currant wines fell on the border between dry and semi-dry wines. Wines contain small amounts of residual sugars, especially fructose, because glucose is the sugar first fermented by most yeast strains [24]. No glucose was found in the wines tested, which indicates that this sugar was entirely used up by microorganisms. The currant and apple wines obtained had a glycerol content of 6.5 g/L and 9 g/L, respectively. The minimum content of glycerol in wines is 5 g/L–8 g/L, but it can reach up to 15 g/L–20 g/L, depending on the initial sugar content, fermentation conditions (temperature), yeast strain, and the addition of sulfur dioxide [29].

### 2.2. Changes in the Polyphenol Content and Antioxidant Activity of Musts and Wines during Simulated Digestion

The presence of polyphenols is an important quality determinant of musts and fruit wines because it affects their organoleptic characteristics, such as color, astringency, and antioxidant properties. The content of these substances in wines depends on how the technological procedures are carried out during their preparation; the key aspect is the degree and time of extraction of polyphenols from skin and fruit tissue during maceration [29]. Musts and wines were analyzed for changes in the content of phenolic compounds, their antioxidant activity, and polyphenol profile under the influence of the digestive process in a simulated human digestive system (Table 2 and Table 3).

More phenolic compounds were found in products obtained from blackcurrant fruit, which is in line with available literature data [11,30]. Interestingly, differences in the total phenolic content (TPC) in must and currant wine, both in the case of non-digested and treated samples, were not statistically significant (Table 2). It can therefore be concluded that the fermentation process does not significantly affect the amount of phenolic compounds contained in blackcurrant wines. Significant differences were only shown after the action of digestive enzymes. It was found that, after each digestion stage of both must and wine types examined, the total polyphenol concentration increased as compared to the previous stage. A similar relationship was demonstrated by Bouayed et al. [31] in their research on the bioavailability of apple polyphenols. Statistically significant differences in the content of polyphenols were also found for must and apple wine after the second and third stages of their digestion, while in TPC wines it was always greater (Table 2). In apples, the dominant group of polyphenols is proanthocyanidins, which are polymers of condensed tannins [32]. Only small amounts of these compounds were found in plasma conjugated with glucuronic acid or sulfonic or methyl groups, which may indicate their passage unchanged into the large intestine, where they are digested with the help of enzymes of intestinal microbiota [21]. The chain length of proanthocyanidins is a factor affecting the degree of degradation of these compounds to phenolic acids, since it has been shown that long-chain molecules can inhibit the action of microbial enzymes [33].

Because proanthocyanidin chains are shorter in wines than in musts [34], the breakdown of high molecular polyphenols occurs more intensively, and therefore larger amounts of polyphenolic acids are released. In must and currant wine, a 3-fold increase in polyphenol content was found after the entire digestion process as compared to the starting material. For apple wine, this increase was more than 4-fold. These results suggest the transformation of high-molecular compounds towards their depolymerization to produce more simpler compounds. These resulting polyphenol derivatives release reactive functional groups into a complex with the Folin–Ciocalteu reagent giving a visible increase in the TPC value. These results are consistent with the literature, confirming the ability of intestinal microbiota to break down polyphenols, including proanthocyanidins and other oligomers, to simpler compounds, mainly phenolic acids [9].

Before digestion, there were no major differences between the antioxidant activity (AOX) of must and wine obtained from a given raw material, with blackcurrant products being stronger antioxidants than those obtained from apples. This is consistent with the research of Borges et al. [35], who demonstrated the high antioxidant activity of blackcurrant fruit, resulting from a significant amount of anthocyanins and vitamin C. Comparing AOX obtained musts and fruit wines with TPC in these tests, one can notice the correlation of both parameters. In studies conducted by Wu et al. [36], the correlation coefficient (R2) between the total polyphenol content and blackcurrant antioxidant activity was 0.96. The correlation coefficient between antioxidant activity and total polyphenol content for musts and currant wines analyzed in this study was 1.00.

Studies have shown that generally during digestion the antioxidant potential increases, especially at the stages of digestion in the small and large intestine (Table 2). Bouayed et al. [31], when analyzing different apple varieties, observed an increase in antioxidant activity after the digestive stage in the small intestine compared to digestion in the stomach; this correlated with the previously determined total polyphenol content at these stages. For some varieties, an increase in the antioxidant activity of the fraction obtained after the first digestion stage, compared to the raw material, was also found. A similar tendency to increase AOX was observed in the tested samples only for wines, whereas for musts, both apple and currant, after the first digestion stage a reduction of the antioxidant potential was shown (Table 2). A significant increase in antioxidant activity was observed for all samples after the digestive stage in the small intestine with participation of the intestinal microflora. Taken with the results of TPC, this suggests that the breakdown of phenolic compounds resulted in the formation of metabolites with higher antioxidant potential.

Since the obtained apple wines were characterized by greater antioxidant activity than musts, it can be assumed that individual phenolic compounds underwent transformation not only during digestion, but also during the fermentation process. During fermentation, yeast metabolizes both the sugars contained in the must and those that are part of the glycoside polyphenols [37]. The ethanol present at the stage of fruit maceration contributes to the leaching of polyphenols from peels and fruit stones. Oxygenation during the pressing and maceration of wines is also important. The access of oxygen significantly affects the transformation of polyphenols in the must, which causes a change in the taste of the wines, especially the taste of astringency and tightness when drinking wine [38]. Phenolic acids are colorless in alcoholic solution, but when they oxidize, they turn yellow. Although they themselves do not bring any taste or smell to a wine, they are precursors of volatile phenolic compounds, the formation of which is related to the activity of microorganisms, e.g., yeasts of the *Brettanomyces* species and bacteria [39]. An important ingredient in fruit musts is tannins, compounds capable of forming stable complexes with proteins and other plant polymers, such as polysaccharides. Tannins reacting with a number of proteins and polysaccharides produce different products. Their reaction with saliva glycoproteins and proline-rich proteins contributes to the tart aftertaste, and contact with protein clarifying agents supports the effect of clarifying wine. Tannins have large molecules formed by the polymerization of basic phenolic compounds. Their configuration and size affect their stability and reactivity in wines. Anthocyanins, responsible for the color of currant or grape skins, are more stable as glycosides than aglycons. Their color depends on pH, structure, and environment. It has been shown that the anthocyanin content in wines decreases during storage and maturation, mainly due to temperature, light, and oxygen [22]. This process negatively affects the quality of wine, leading to a loss of the desired color. It is worth emphasizing, however, that anthocyanins can form complexes with tannins, which results in the creation of new, more stable compounds. They also react with compounds containing an alpha-dicarbonyl group, such as diacetyl [22]. Even before the start of alcoholic fermentation, condensation reactions of some compounds from the group of anthocyanins, catechins, and proanthocyanidins occur, causing the formation of new polymers in the nature of dyes [40].

### 2.3. Changes in the Polyphenol Profile of Musts and Wines during Simulated Digestion

Analysis of the phenolic compound profile in the examined musts and apple wines confirmed the presence of chlorogenic acid, catechin, and phloridzin (Table 3). The literature presents a number of results on the profile of phenolic compounds in apples. De Paepe et al. [12] analyzed the skin and flesh of apples of the Rubin variety. The skin was found to contain numerous compounds from the group of phenolic acids, flavonols, flavan-3-ols, and dihydrochalcones. In the pulp, the content of these substances was several dozen times lower, and a significant amount contained chlorogenic acid, catechin and epicatechin, phloridzin, p-coumaric acid, and procyanidin B2. Polyphenol content in apples of the Rubin variety was also observed by Łata et al. [32]. Considering the whole fruit, without division into skin and flesh, chlorogenic acid was the substance occurring in the highest amount, which is consistent with the results obtained. Flavan-3-ols were another important group of compounds, mainly catechin, followed by rutin and phloridzin. As with the results obtained in this study, no other phenolic acids, such as caffeic acid, nor quercetin and its derivatives were found. On the contrary, Wojdyło et al. [41] determined the high content of oligomeric procyanidins, chlorogenic acid, quercetin glycosides, catechins, and phloridzin in the Rubin variety. Differences in the profile of phenolic compounds within a given cultivar result from the variability of environmental factors. Even in the same areas, but in different years, fruits have different qualitative and quantitative composition of these compounds [32].

The described compounds undergo various transformations during digestion in the human digestive system. Flavan-3-ol monomers such as catechin are absorbed through biological membranes without prior hydrolysis [42]. After absorption, these compounds are conjugated with glucuronic acid and the methoxy group in intestinal epithelial cells and only a small part is excreted unchanged [43]. Bell et al. [44] showed that the increase in the amount of catechin in plasma after consumption of traditional and de-alcoholized wines does not depend on the presence of alcohol in the drink. At low pH, catechin is transformed into colorless compounds, whereas at higher pH, catechin is transformed into yellow compounds [45]. Compared to the catechin content in non-digestible musts and apple wines, an increase in the amount of catechin was observed after the second stage of the process. This may indicate the distribution of flavan-3-ol oligomers to monomers as a result of digestive enzymes and the high pH that prevails in the small intestine. The decrease in catechin concentration at the stage of the large intestine indicates further transformation of this compound, including its degradation to simpler compounds by the enzymes of intestinal bacteria. This is confirmed by the literature indicating the transformation of catechin and epicatechin into phenylpropionic acid and valerolactones [46].

The presence of chlorogenic acid was found in most of the analyzed samples, with the exception of must and currant wine and the third stage of digesting musts and apple wines. Chlorogenic acid present in must and apple wine undergoes partial hydrolysis under conditions in the stomach, which causes an initial reduction in its concentration (stage I). At the same time, under the influence of further digestion, large molecules of polymeric polyphenols are degraded to simpler compounds, including chlorogenic acid, which is visible in a higher concentration at stage II of digestion [47]. After incubation of apple must and apple wines with the intestinal microbiota, no further chlorogenic acid was found, since it is broken down by bacterial esterases to quinic and caffeic acids, which are further transformed into phenylpropionic acids, and after decarboxylation into phenylacetic acids [9].

Phloridzin is a glycoside of phloretin—a compound from the dihydrochalcone group. Phloridzin was present in small amounts in all the analyzed samples, and its content in must and apple and currant wines was comparable. The reduction in phloridzin content during digestion is caused by its enzymatic hydrolysis to phloretin [48], and additionally in the large intestine under the influence of intestinal microbiota by the decay to phloroglucinol and 3-(4-hydroxyphenyl) propionic acid [49,50].

In the conducted research, clear discrepancies in the polyphenol profile were observed between apples and blackcurrants. The main difference is due to the presence of anthocyanins in blackcurrants such as callistephin (pelargonidin-3-glucoside) and keracyanin (cyanidin-3-rutinoside). Callistephin is a dye found in red flower petals and fruit peels such as raspberries, strawberries, cranberries, grapes, and currants. Slimestad and Solheim [51], Wu et al. [36], and Borges et al. [35] also found the presence of these anthocyanins in blackcurrant, indicating keracyanin as one of the dominant color compounds in these fruits. They also showed that the content of anthocyanin rutinosides is higher than that of glucosides, which is consistent with the results obtained in this work. Significantly lower anthocyanin concentrations in currant wines compared to musts were observed (Table 3). The likely cause may be partial hydrolysis of glycosides during yeast fermentation [40]. Oxygenation of musts during maceration also degrades the amount of glycosides [36]. Digestion studies of anthocyanins found in pomegranate juice, conducted by Pérez-Vincente et al. [52], showed a significant decrease in the content of these compounds after the digestive stage in the small intestine with the participation of pancreatin and bile. Matsumoto et al. [53] conducted in vivo studies that showed that some anthocyanin glycosides, such as keracyanin, are absorbed directly from the gastrointestinal tract into the bloodstream without prior hydrolysis, while Nielsen et al. [54] found that blackcurrant anthocyanin rutinosides are absorbed to a greater extent than glucosides, notably that the structure of aglycon did not affect these processes. It is known that in low pH environments, such as those in the stomach, anthocyanins occur in the form of a red oxonium ion (flavyl cation). The observed reduction in the amount of anthocyanins after the second stage of digestion may therefore result from the transformation of the flavyl cation into colorless chalcones that are stable at pH 7.0–7.5. Anthocyanin degradation to other colorless compounds is also possible. Both in the must and currant wine analyzed, a reduction in keracyanin content after the first digestion stage was shown by 48% in comparison with the initial sample. After the digestion stage in the conditions of the small intestine, this compound was no longer found, indicating the instability of keracyanin in an alkaline environment. In the case of callistephin, the content in musts and currant wines after the gastric digestion stage was 62% and 72% of the initial value, respectively, and decreased in the subsequent digestion stages. It is known that, in the large intestine, anthocyanin glycosides can be hydrolyzed by bacterial enzymes to aglycons, followed by simpler compounds such as protocatechuic acid, which would explain the reduction in callistephin content after this digestion stage [55].

## 3. Materials and Methods

The research material consisted of apples of the Rubin variety, originating from the pomological orchard of the University of Agriculture in Krakow, Poland, and blackcurrant fruit from plantations in the Krakow area.

The apples and currants were washed, the apples were additionally crushed (with a laboratory crusher, Robix, Veszprem, Hungary), and then the must was pressed out of the fruit (wooden manual basket press, dimensions: φ = 25 cm, h = 36 cm). The musts were subjected to correction of the extract (up to 20 °Blg) and acidity (for apple must 4.5 g/L–5.0 g/L, for currant must 7 g/L), and then inoculated with rehydrated Erbslöh Oenoferm^®^ InterDry F3 yeast (Marxam, Krakow, Poland) in the amount of 0.3 g/L. Fermentation of apple must was carried out at 20 °C ± 2 °C for thirty days, and currant must for fifty days, and then the wines were aged at 5 °C (cold room with adjustable temperature) for two months. Full anaerobic conditions were ensured; the wines were aged in bottles sealed with rubber stoppers and secured with parafilm. The access of light to the wines was also completely eliminated (darkened, closed cold store room).

The musts and wines obtained from them served as the material for analysis and were used in the simulated digestive process. All tests and analyses were performed in a minimum of three physical replicates.

### 3.1. Determination of Extract Content, Alcohol, Total Acidity, and Volatile Acidity

Determinations of total extract, ethyl alcohol, and acidity were carried out in accordance with the methods recommended by the International Organization of Vine and Wine (OIV) [56]. The total extract and ethanol content were determined using distillation methods with pycnometric density determination. Briefly, the wine sample (100 mL) was distilled, the distillate was made up to 100 mL with distilled water, and its density was measured using a pycnometer. The alcohol content in the wine was read from the tables. Then, the distillation residue was quantitatively transferred to a volumetric flask, made up to 100 mL with distilled water, and the extract content was measured with an Abbe refractometer (PZO S.A., Warsaw, Poland). Total acidity was determined by potentiometric titration with 0.1 M NaOH. The results were expressed in g of malic acid/L. The volatile acidity analysis was performed by steam distillation of the wine followed by titration of the distillate with 0.1 M NaOH. The results were expressed in g of acetic acid/L.

### 3.2. Procedure of In Vitro Digestion

Musts and extracts (0.5 mL) were acidified to pH = 2 with HCl (0.5 M, POCh, Gliwice, Poland) into screw-capped vials. Then were added 0.75 mL of pepsin solution (62.6 mg of pepsin, EC 3.4.23.1, with activity 3440 U/mg, Sigma-Aldrich, St. Louis, MO, USA) dissolved in 20 mL of 0.1 M HCl (POCh) and redistilled water (obtained by ultrafiltration, Simplicity Millipore, Temecula, CA, USA) to a total volume of 3 mL. After thorough mixing, the test tubes were incubated in a water bath at 37 °C for 2 h. At this stage (digestion stage I, stomach), some samples were centrifuged (880× *g*, 10 min) and the supernatants forwarded for further analysis.

NaHCO_3_ (POCh) in an amount providing pH = 7.0 was added to the remaining samples, followed by 0.375 mL of pancreatin and bile solution. The solution was prepared by mixing 66.7 mg of pancreatin, (EC 232-468-9, with activity 8 × USP, Sigma-Aldrich) and 833.3 mg of bile (EC 232-369-0, Sigma-Aldrich), which was then dissolved in 10 mL of 0.1 M NaHCO_3_. The sample volume was adjusted to 5 mL with redistilled water. Vials were incubated in a water bath (37 °C, 4 h) and centrifuged (1380× *g*, 10 min). Then, some samples were centrifuged and the obtained supernatants were retained for further analysis (digestion stage II, small intestine).

Under anaerobic conditions, intestinal bacteria of documented human origin (*Bifidobacterium catenulatum*, *Escherichia coli*, *Ruminococcus gauvreauii*, *Enterococcus caccae*, *Lactobacillus* sp.; Leibniz Institut DSMZ—Deutsche Sammlung von Mikroorganismen und Zellkulturen, Braunschweig, Germany), an inoculum containing 10^6^ bacterial cells (with equal participation of individual species) was prepared in 1 mL of the mixture. After being centrifuged (4000× *g*–5000× *g*, 20 min), the supernatant was discarded leaving the bacterial pellet. Under anaerobic conditions, samples after previous digestion (supernatant from the small intestine stage) were added to the bacterial pellet, mixed, saturated with inert gas (N_2_), and incubated at 37 °C for 16 h (interaction of intestinal microbial enzymes). The samples were then centrifuged and the supernatant obtained was retained for further analysis (digestion stage III, large intestine).

### 3.3. Antioxidant Activity (AOX)

Antioxidant activity (AOX) was determined according to the method described by Tarko et al. [57], using an active cation radical of 2,2′-azino-bis (3-ethylbenzothiazoline-6-sulfonic) acid (ABTS, Sigma-Aldrich). AOX was calculated on the basis of a calibration curve, prepared each time for synthetic vitamin E ((±)-6-hydroxy-2,5,7,8-tetramethylchromane-2-carboxylic acid, Trolox, Sigma-Aldrich) and expressed as mg of Trolox/100 mL.

### 3.4. Total Polyphenol Content (TPC)

Total polyphenol content (TPC) in musts, along with all obtained fractions of simulated digestion, was determined by the method described previously in detail by Tarko et al. [57], i.e., by the Folin–Ciocalteu method. The results were expressed as mg of (+)-catechin/100 mL.

### 3.5. Analysis of Phenolic Compound Profiles

Prior to analysis, the samples were filtered through a nylon syringe filter (0.45 µm, Chemland, Stargard Szczecinski, Poland). Analysis of the polyphenol profile was carried out using high-performance liquid chromatography (HPLC, Shimadzu, Kyoto, Japan) equipped with a DAD detector. A Synergi Fusion RP-80A column 150 mm × 4.6 mm (4 µm) (Phenomenex, Torrance, CA, USA), thermostated at 30 °C temperature, was used for all analyses. Acetonitrile (POCh) and 2.5% aqueous solution of acetic acid (POCh) were used as a mobile phase. The detailed gradient program and detection wavelengths were described in detail by Tarko et al. [58].

For quantitative analyses, calibration curves were prepared for the following standards: ferulic acid, caffeic acid, chlorogenic acid, gallic acid, hippuric acid, p-coumaric acid, protocatechuic acid, ellagic acid, (+)-catechin, quercetin, resveratrol, kaempferol (Sigma-Aldrich), phloridzin, (−)-epicatechin, procyanidins B1 and B2, cyanidin-3-*O*-galactoside, cyanidin-3-*O*-sambubioside, cyanidin-3-*O*-arabinoside cyanidin-3-*O*-glucoside, delphinidin-3-*O*-glucoside, quercetin-3-*O*-rutinoside, quercetin-3-*O*-glucoside, pelargonidin-3-*O*-glucoside, and peonidin-3-glucoside (Extrasynthese, Genay, France). Compounds were identified by comparing the retention time of individual peaks and UV spectra with those of standards (using the spectrum library for the standards). Polyphenols not detected in any experimental variants were not included in the tables.

### 3.6. Determination of Sugar and Glycerol Concentration (HPLC)

The concentration of sugars and glycerol was determined by the HPLC method. The analysis of the sugar profile was conducted with the Shimadzu (Kyoto, Japan) NEXERA XR apparatus with an RF-20A refractometric detector. The separation was conducted with an Asahipak NH2P-50 4.6 mm × 250 mm Shodex column (Showa Denko Europe, München, Germany) thermostated at 30 °C. The mobile phase consisted of an acetonitrile aqueous solution (70%), and the isocratic elution program (0.8 mL/min) lasted 16 min.

Quantitative determinations were made using calibration curves prepared for appropriate standards, i.e., glucose, fructose, sucrose, and glycerol (POCh, Gliwice, Poland).

### 3.7. Statistical Analysis

A minimum of three repetitions of the analysis was conducted, and the results are shown as the arithmetic mean with standard deviation (±SD). Statistical analysis was performed using InStat v. 3.01 (GraphPad Software Inc., San Diego, CA, USA). A single-factor analysis of variance (ANOVA) with post hoc Tukey’s test was applied to determine the significance of differences between means. The Kolmogorov–Smirnov test was carried out to assess the normality of the distribution.

## 4. Conclusions

Blackcurrant wines have a higher total polyphenol content and antioxidant activity compared to apple wines. The fermentation process did not significantly affect the total polyphenol content in the obtained wines compared to the original must. The profile of phenolic compounds depends on the type of fruit from which musts and wines are obtained. In apple wine, the presence of chlorogenic acid, catechin, and phloridzin was found, whereas blackcurrant was dominated by compounds from the anthocyanin group, such as keracyanin and callistephin. In addition, the antioxidant activity of fruit wines is positively correlated with the content of total polyphenols. As a result of digestion, the amount of phenolic compounds increased, with the largest increase in polyphenol content observed after the digestion stage simulating the conditions in the small intestine. As a result of the action of intestinal bacterial enzymes, polyphenol compounds were transformed into simpler components with higher antioxidant potential.

## Figures and Tables

**Table 1:** Characteristics of wines.

Wine Type	Apple Wine	Blackcurrant Wine
Total extract content (g/L)	20.48 ± 0.66 ^a^	21.53 ± 0.81 ^b^
Ethanol content (% vol.)	12.70 ± 0.07 ^a^	12.17 ± 0.23 ^b^
Total acidity (g of malic acid/L)	4.43 ± 0.05 ^a^	6.80 ± 0.09 ^b^
Volatile acidity (g of acetic acid/L)	0.37 ± 0.02 ^a^	0.35 ± 0.02 ^a^
Glucose content (g/L)	0 ^a^	0 ^a^
Fructose content (g/L)	2.52 ± 0.53 ^a^	10.64 ± 0.93 ^b^
Glycerol content (g/L)	9.38 ± 0.33 ^a^	6.54 ± 0.15 ^b^

a, b: means marked with the same letter in a row are not significantly different at *p* < 0.05, *n* = 3.

**Table 2:** Antioxidant activity (AOX) and total phenolic content (TPC) in musts and wines at particular stages of digestion.

Sample		Stages of Digestion			
		Undigested	Stage I (Stomach)	Stage II (Small Intestine)	Stage III (Large Intestine)
AOX *	Apple must	52.68 ± 0.01 ^i^	48.79 ± 12.49 ^a^	191.18 ± 21.96 ^d,f^	226.29 ± 24.04 ^g^
	Apple wine	62.68 ± 2.42 ^i^	98.43 ± 8.17 ^b^	210.90 ± 14.54 ^e,f^	239.97 ± 10.38 ^g^
	Blackcurrant must	152.67 ± 0.19 ^h^	95.89 ± 12.53 ^b^	196.32 ± 14.15 ^f^	239.70 ± 13.98 ^g^
	Blackcurrant wine	130.75 ± 0.11 ^h^	137.30 ± 9.89 ^c^	234.66 ± 8.65 ^g^	254.67 ± 15.05 ^h^
TPC **	Apple must	30.92 ± 0.01 ^i^	34.79 ± 8.75 ^a,h^	68.81 ± 6.55 ^c^	99.68 ± 8.68 ^e^
	Apple wine	22.27 ± 0.20 ^i^	36.49 ± 2.16 ^a,h^	82.66 ± 6.62 ^d^	110.68 ± 17.14 ^f^
	Blackcurrant must	42.00 ± 0.00 ^h^	52.64 ± 4.86 ^b^	100.97 ± 12.56 ^e,f^	133.57 ± 17.30 ^g^
	Blackcurrant wine	41.73 ± 1.98 ^h^	50.43 ± 2.55 ^b,h^	102.02 ± 7.85 ^e,f^	128.46 ± 8.92 ^g^

a–i: means related to the tested parameter marked with the same letter are not significantly different at *p* < 0.05, *n* = 3; * [mg of Trolox/100 mL]; ** [mg of catechin/100 mL].

**Table 3:** Phenolic compound concentrations [mg/L] in musts and wines at particular stages of digestion.

Sample		Stages of Digestion			
		Undigested	Stage I (Stomach)	Stage II (Small Intestine)	Stage III (Large Intestine)
Chlorogenic acid	Apple must	12.54 ± 0.40 ^d^	7.52 ± 0.92 ^a^	16.96 ± 1.64 ^c^	0 ^b^
	Apple wine	17.52 ± 0.27 ^c^	8.30 ± 0.74 ^a^	13.56 ± 2.74 ^d^	0 ^b^
	Blackcurrant must	0 ^b^	0.30 ± 0.03 ^b^	0.34 ± 0.07 ^b^	0.26 ± 0.03 ^b^
	Blackcurrant wine	0 ^b^	0.29 ± 0.02 ^b^	0.20 ± 0.12 ^b^	0.14 ± 0.05 ^b^
Catechin	Apple must	4.20 ± 0.70 ^e^	1.89 ± 0.14 ^a^	6.32 ± 0.70 ^d^	1.05 ± 0.56 ^b^
	Apple wine	3.22 ± 0.05 ^f^	1.11 ± 0.29 ^b^	6.86 ± 0.63 ^d^	2.14 ± 0.48 ^a^
	Blackcurrant must	0 ^c^	0 ^c^	0 ^c^	0 ^c^
	Blackcurrant wine	0 ^c^	0 ^c^	0 ^c^	0 ^c^
Phloridzin	Apple must	0.11 ± 0.00 ^f^	2.34 ± 0.16 ^a^	1.36 ± 0.07 ^b^	0.59 ± 0.18 ^d^
	Apple wine	0.05 ± 0.00 ^f^	2.34 ± 0.20 ^a^	1.32 ± 0.12 ^b^	0.79 ± 0.05 ^d,e^
	Blackcurrant must	0.13 ± 0.01 ^f^	2.55 ± 0.06 ^a^	1.23 ± 0.20 ^b^	0.53 ± 0.19 ^d^
	Blackcurrant wine	0.08 ± 0.03 ^f^	2.33 ± 0.17 ^a^	1.96 ± 0.50 ^c^	0.78 ± 0.23 ^d,e^
Keracyanin	Apple must	0 ^a^	0 ^a^	0 ^a^	0 ^a^
	Apple wine	0 ^a^	0 ^a^	0 ^a^	0 ^a^
	Blackcurrant must	256.62 ± 10.02 ^d^	133.44 ± 8.08 ^b^	0 ^a^	0 ^a^
	Blackcurrant wine	76.61 ± 12.68 ^e^	39.72 ± 4.35 ^c^	0 ^a^	0 ^a^
Callistephin	Apple must	0 ^a^	0 ^a^	0 ^a^	0 ^a^
	Apple wine	0 ^a^	0 ^a^	0 ^a^	0 ^a^
	Blackcurrant must	113.89 ± 2.19 ^g^	70.53 ± 3.42 ^b^	29.61 ± 3.32 ^c^	16.93 ± 3.87 ^e^
	Blackcurrant wine	37.58 ± 3.15 ^h^	27.10 ± 3.76 ^c^	7.71 ± 1.58 ^d^	3.98 ± 0.86 ^f^

a–h: means related to the particular phenolic compound marked with the same letter are not significantly different at *p* < 0.05, *n* = 3.
